# Regenerative medicine in China: main progress in different fields

**DOI:** 10.1186/s40779-016-0096-z

**Published:** 2016-08-19

**Authors:** Biao Cheng, Shu-liang Lu, Xiao-bing Fu

**Affiliations:** 1 Medical College of PLA, General Hospital of PLA, College of Life Sciences, Beijing, 100853 China; 2 The Key Laboratory of Trauma Treatment & Tissue Repair of Tropical Area of PLA, Guangzhou, 510010 China; 3 Shanghai Burns Institute, Ruijing Hospital, Shanghai Jiaotong University, Shanghai, 200025 China

**Keywords:** Regenerative medicine, Stem cells, Tissue engineering, China

## Abstract

Regenerative medicine (RM) is an emerging interdisciplinary field of research and China has developed the research quickly and impressed the world with numerous research findings in stem cells, tissue engineering, active molecules and gene therapy. Important directions are induced differentiation of induced pluripotent stem and embryo stem cells as well as somatic stem cell differentiation potential and their application in trauma, burns, diseases of aging and nerve regeneration. The products ActivSkin and bone repair scaffolds have been approved and are applied in the clinic, and similar products are being studied. About 10 engineered growth-factor drugs for repair and regeneration have been approved and are used in the clinic. Gene therapy, therapeutic cloning and xenotransplantation are some of the strategies being studied. However, China needs to develop standards, regulations and management practices suitable for the healthy development of RM. Aspects that should be strengthened include sound administrative systems, laws, and technical specifications and guidelines; conservation of stem cell resources; emphasis on training and retention of talented stem cell researchers; and reasonable allocation of resources, diversification of investment and breakthroughs in key areas. Finally, broad and deep international cooperation is necessary.

**Electronic supplementary material:**

The online version of this article (doi:10.1186/s40779-016-0096-z) contains supplementary material, which is available to authorized users.

## Background

Since 2002, major foreign media has paid much attention to the progress of Regenerative Medicine (RM) in China included the *Wall Street Journal*, *New Scientist* and *Times*, and *Journal of Applied Behavioral Analysis* [1] (www.iias.nl/iiasn/29/IIASNL29_49.pdf). In 2009, many articles were published in *Nature* and the subjournal *Nature Reviews Molecular Cell Biology* [2, 3], *Cell* and subjournal *Cell Stem Cell* [4], *New England Journal of Medicine* [5], and *Regenerative Medicine* and *Science* [6–8]. Many media has noted the significant progress in China and interpreted China’s policies on stem cells, tissue engineering and related areas. It noted that the Chinese government has invested a large amount of funding in RM represented by stem cell study, tissue engineering and clinical translational studies and the country has made great progress. Some reports indicated that the Chinese government will enhance investment in RM and has been active in recruiting well-trained researchers in the 21st century and that China is leading the area in some aspects. However, China still faces challenges in innovation, translational application, regulation, governance and management.

## Progress in main fields of regenerative medicine

### Stem cells

#### Overview

Chinese researchers started to pay much attention to stem cells during the 1980s. In China, it is prohibited to conduct reproductive cloning, utilize a human embryo beyond day 14, fuse human and non-human gametes and implant research embryos into a human or animal uterus. The government has greatly invested in stem cell research concentrated in several key labs in Beijing and Shanghai. In 2001, the Chinese Academy of Sciences (CAS) established a key lab of stem cell biology, which was followed by the establishment of a stem cell research network composed of the Shanghai Life Science Institute of CAS, Guangzhou Institutes of Biomedicine and Health, Biology Physics Institute, Zoology Institute, Genetics and Development Institute and Kunming Zoology Institute. (1) Key Laboratory of Stem Cell Biology, Shanghai Institutes for Biological Sciences, Chinese Academy of Sciences. Partners are Shanghai Jiaotong University School of Medicine, Shanghai Xinhua Hospital, Changzhou City First People’s Hospital, the Third Hospital Affiliated to Suzhou University, established a biomedical translational research base. The research focuses on establishing embryonic stem cell lines, isolating tissue stem cells, studying their stemness and differentiation induction, and stem cell immunology. The main focus is on regulating the differentiation of stem cells, aiming to solve several major problems in the clinical application of stem cells and developing protocols to derive inducible pluripotent stem (iPS) to further understand their roles in disease development. (2) Guangzhou Institutes of Biomedicine and Health, Chinese Academy of Sciences—South China Institute for Stem Cell Biology and Regenerative Medicine, Key Laboratory of Regenerative Biology. This is first international demonstrative base of technology collaboration. Guangdong International Technology Collaboration Demonstrative Base of Stem Cell and Regenerative Medicine. Cooperation institutions are Korea Stem Cell Research Center/YonSei University College of Medicine, Faculty of Medicine, and the Chinese University of Hong Kong. Research Areas are chemical biology for stem cell, stem cell physiology, therapeutic, differentiation, molecular diagnoses and self renew the mechanism of induced pluripotent stem cell and its clinical application. (3) The Institute of Biophysics, Chinese Academy of Sciences. Research areas are biology of embryonic stem cell, pluripotent stem cells (PSCs), the function and regulation of neural stem cells in the mammalian brains; Institute of Zoology, Chinese Academy of Sciences—the Research Center of Stem Cells and Regenerative Medicine. The Chinese-French Laboratory of Biology of Embryonic Cells of Mammalians (LABIOCEM) combined with the French National Institute for Agricultural Research focusing on stem cells and iPS cells of domestic animals, the mechanisms of cloning and therapeutic cloning, which markedly improved the efficiency of animal cloning; The Institute of Genetics and Developmental Biology of the Chinese Academy of Sciences and Nanjing Drum Tower Hospital (the affiliated hospital of Nanjing University Medical School) established the Nanjing Stem Cells and Biomaterials Research Center, focused on stem cell 3D culture and self-renewal regulatory network, stem cell and biomedical materials, tissue regeneration and wound healing and stem cell translational medicine. (4) The Kunming Institute of Zoology, the Chinese Academy of Sciences, cooperation with Yunnan provincial government established the Key Laboratory of Animal Reproductive Biology focused on rhesus monkey embryonic stem cell self-renewal mechanism with primate animal disease models, research stem cell pharmacology, and promoting Chinese clinical stem cell therapy. Other famous universities and institutes have established stem cell institutes or centers with or without international cooperation (Table 1). Thus, China is close to the global advanced-research level of embryo stem cells and other stem cells [9].

**Table 1:** Main stem cell research institutes in China

Time	Name	Composition
2001.01	Peking University Stem Cell Research Center	Peking University
2013.07	Peking University Center for Craniofacial Stem Cell Research and Regeneration	
2001.02	Union Stem Cell and Gene Engineering	Institute of Hematology and Blood Diseases Hospital, Chinese Academy of Medical Sciences and Peking Union Medical College
2002.01	Institute of Reproductive and Stem Cell Engineering, Central South University	Central South University
2004	National Engineering Research Center of Human Stem Cells	
2005	Key Laboratory of Human Stem Cells and Reproductive Engineering	
2003.01	Center for Stem Cell Biology and Tissue Engineering Sun Yat-Sen University	Sun Yat-Sen University
2008.10	Med-X-Renji Hospital Clinic Stem Cell Research Center	Med-X Research Institute of Shanghai Jiao Tong University, Renji Hospital
2011.03	Center of Stem Cells and Regenerative Medicine, Tsinghua University	Tsinghua University
2012.01	Research Center of Stem Cell and Developmental Biology, Zhejiang University	Zhejiang University
2012.04	Sino-US Research Center of Regenerative and Translational Medicine	Institute for Regenerative Medicine, Wake Forest University, Key Laboratory of Neuroregeneration, Nantong University
2012.06	Sino-US Research Center of Stem Cell	Tongji University, California Institute for Regenerative Medicine
2012.12	Southern China Center for Stem Cell and Regenerative Medicine	Academy of Military Medical Sciences, Guangdong Provincial Department of Science and Technology

#### Stem cell banks

In 2007, the Ministry of Science and Technology (MST) established 4 stem cell banks covering the north, south and east of China. The banks support each other with their own technological advantages and are expected to create a platform for 3 or 4 key technologies of stem cells (http://www.973.gov.cn/ReadItem.aspx?itemid=1141). (1) Northern Stem Cell Bank and Parthenogenetic hESCs Lines Technology Platform. To establish key technology of clinical-grade human embryonic stem cells and human parthenogenetic embryonic stem cell bank; to create their own stem cells and collect a variety of resources; supply stem cell materials, information, knowledge and technology services; and support for research institutions. (2) Southern Stem Cell Bank and Diseases Stem Cell Lines Technology Platform. To establish disease stem cell lines and hpESC lines, and parthenogenetic technology platform in hESC lines, complete common stem cell culture technique and operating instruction. (3) Chinese Academy of Sciences, Stem Cell Bank and Stem Cell Gene Manipulation Technology Platform. To establish, collect, identify, store and provide stem cells and relevant technique and materials, and improve China stem cell resources (especially human embryonic stem cells), and promote China stem cell research and international academic exchange. (4) Eastern China Stem Cell Bank and Clinical Grade hESC Lines Technology Platform. To take charge of the National Stem Cell bank websites and databases and stem cell bank management and coordination. To establish clinical-grade stem cell lines and non-animal ingredients hESC lines, offer a variety of standardized stem cells, and provide stem cell technical consultation and training. In 2002, the information network for hematopoietic stem cell donators was released formally online. In 2010, the Ministry of Health (MH) planned to establish 10 hematopoietic stem cell banks.

#### Basic stem cell studies

Before 2007, basic stem cell studies in China concentrated on bone-marrow and embryo stem cells [10]. Since then, China was gradually moving toward the top position in basic stem cell research [11]. Liu et al. [12] reported the first iPS cell line from rhesus monkey in *Cell Stem Cell*. In 2009, Zou et al. [13] first isolated reproductive stem cells and cultured reproductive stem cell strains capable of long-term self-renewal. Li et al. [14] first cultivated a mouse by using iPS cells. This was the first proof of the totipotency of iPS cells. This finding was elected by *Times* as one of the Global Top 10 Biomedicine Advances. The journal believed that “this study is a symbol of a major step forward of stem cell research”. In early 2010, Esteban et al. [15] increased the iPS induction efficiency by 10-fold by adding vitamin C. The *Proceedings of the National Academy of Science USA* published the finding the new function of the stem cell factor receptor C-KIT and application in translational medicine [16]. Chinese researchers published their success in creating cell lines from androgenetic haploid embryonic stem cells, a breakthrough in embryo stem cell research [17]. In 2013, Hou et al. [18] used a small molecular compound to induce the reprogramming of somatic cells into multipotent stem cells. In 2013, *Cell* published a special “*Spotlight on China*” edition that highlighted the rapid development of immunology study in China and in particular, positively commented on the immunology aspects of applying stem cells for clinical treatment [19] (http://www.cell.com/spotlightonchina).

#### Therapeutic applications

The practice of stem cell clinical trials or treatment in China dates back to bone-marrow transplantation in the 1960s, really the transplantation of stem cells in bone marrow. China declared the legality of stem cell treatment as a medical technique following the United Kingdom and the United States.

For therapeutic applications, Zhu and colleagues treated a woman who had chopsticks inserted into her brain from the eyes, which resulted in frontal cerebral-cortex injury. The authors cultivated the brain tissue attached to the chopsticks and were interested in stem cell–motivated self-repair [20]. In 2009, Sheng et al. [21] regenerated sweat glands by using bone-marrow mesenchymal stem cells (MSCs). This technology has been applied in more than 30 cases with follow-up for more than four years. In 2013, umbilical-cord MSC transplantation was performed in patients with post-traumatic brain syndrome. A total of 40 patients with post-traumatic brain syndrome were randomized to receive stem cell or control treatment. Umbilical-cord MSC transplantation improved the neurological function of the patients. However, these results need to be confirmed by prospective, randomized, multi-center, large clinical studies [22]. At the website of the State Food and Drug Administration (SFDA) (http://www.sda.gov.cn/WS01/CL0001/) (date of search: 2013-08-01), a few stem cell–related products have been approved for clinical trials (Table 2).

**Table 2:** Stem cells products approved for clinic pre-clinic trial by the SFDA

Accept No.	Generic name	Usage	Date	Unit	Status
X0400586	Bone marrow mesenchymal stem cells	Injection	2004.02	The foundation of the Chinese academy of sciences institute of medicine	Approved
CSL20020071	Human recombinant stem cell factor for injection	Injection	2003.05	The Second Military Medical University	Approved
X0408234	Mesenchymal stem cells in myocardial infarction for injection	Injection	2005.01	Beijing Yuanhefa Biotechnology	Approved
X0407487	Autologous bone marrow mesenchymal stem cells for injection	Injection	2004.12	Institute of transfusion medicine, Academy of Military Medical Science of PLA	Approved
CSL01037	Recombinant stem cell factor for injection	Injection	2001.09	Institute of transfusion medicine, Academy of Military Medical Science of PLA	Approved
X0404120	Umbilical cord blood nuclear progenitor cell for injection	Injection	2004.07	Institute of transfusion medicine, Academy of Military Medical Science of PLA	Approved
X0404119	Umbilical cord blood red blood cell precursors for injection	Injection	2004.08	Institute of transfusion medicine, Academy of Military Medical Science of PLA	Approved

**Fig. 1:**
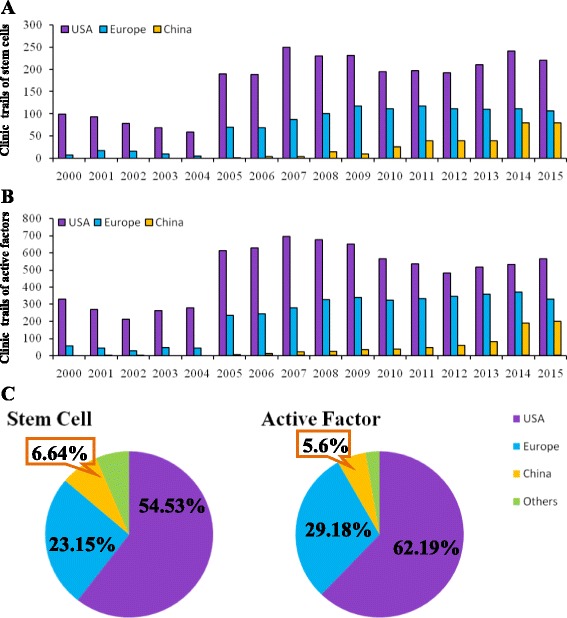
Clinical trials about stem cells and active factors in China, United States and European countries (www.clinicaltrials.gov/). **a** Stem cells; **b** Active factors; **c** Stem cells and active factors

Before 2005, China had no stem cell therapies and activity factor clinical trials registered at ClinicalTrials.gov (http://www.clinicaltrials.gov/, Fig. 1) (Additional files 1 and 2). After then, Chinese scholars have been paying increasing attention to this issue and the projects registered have been increasing annually, reaching 138 in 2012. In 2013, at the time of our search (August 20, 2013), the number of projects registered reached 28.

#### Comparison with research-advanced countries on stem cells

**Fig. 2:**
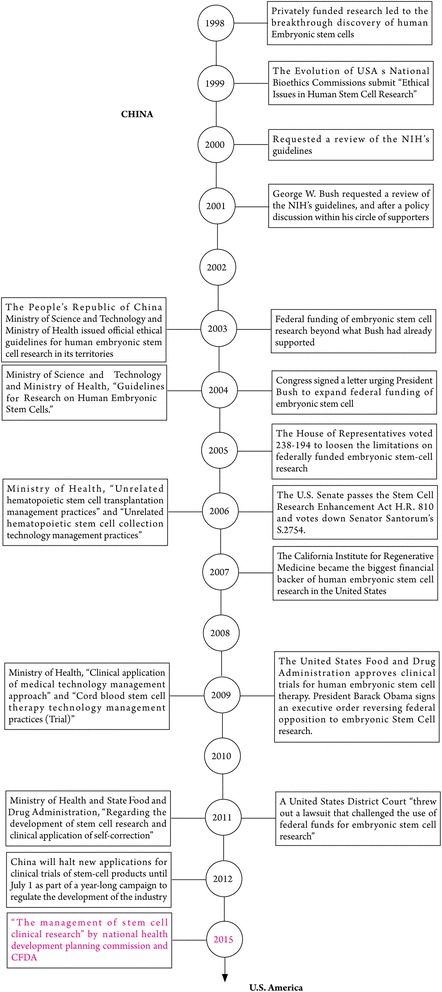
Comparison of stem cell research policies between China and the United States and related organizations

The United States maintains a leading position in stem cell research, for a significant difference between the United States and China (Fig. 2). The United States has implemented strict management since 1998. The CAS, MST and MH in China started to make laws and policies associated with stem cells in 2003. However, China has improved a variety of policies so far and hopes for real enforcement. In countries such as the United Kingdom and Sweden, collecting human embryonic stem cells for application is relatively free but is more stringent in other countries. In all countries, human cloning and destruction of human embryos are prohibited.

In the field of stem cell drug development and clinical trials, before December 31, 2009, the US Food and Drug Administration (FDA) approved 2,980 projects on stem cell therapy. In 2009, it allowed Geron, a biotech company in California, to perform the world’s first clinical trial on human embryonic stem cells, which was a milestone in the history of medicine (http://www.biotech.org.cn/information/85021). Subsequently, another clinical trial of human embryonic stem cells, performed by Advanced Cell Technology, was approved by the US FDA. In 2010, the United Kingdom approved its first stem cell trial in humans. In recent years, 3 stem cell therapeutic agents have been approved in South Korea. A stem cell product, Prochymal (manufactured by Osiris), was approved by Health Canada in May 2012. This product was the first non-prescribed MSC agent approved by a developed country for treating acute graft-versus-host disease worldwide. In addition, the Australian Therapeutic Goods Administration approved the production and supply of an autologous mesenchymal precursor cell product, Mesoblast. About 3,500 clinical trials involving somatic stem cells are registered by the US National Institutes of Health. More than 2,000 clinical trials of stem cell therapy are ongoing. Most of these clinical trials are phase I to II. These clinical trials were performed mainly in the United States and Europe. In China, stem cell therapy is restricted, and the hospitals need to file with the regulatory authorities to perform studies. In 2012, the project “Mesenchymal stem cells in myocardial infarction injection”, involving one of the three stem cell drugs improved for clinical trials in China 6 years ago, completed its phase I clinical trials. Before this, the SFDA approved two stem cell drugs for clinical trials: “primitive bone-marrow MSCs” and “autologous bone-marrow MSC injection”.

For stem cell drugs, China has lagged behind other countries in strong stem cell research capability. Because of lack of detailed rules in laws, the stem cell market is in chaos driven by financial interests. The chaos in the area of stem cell therapy in China was reported in the *New England Journal of Medicine* [5] and *Nature* [23, 24] in 2009 and 2010. The articles indicated concern about the safety of stem cell therapy in China. The MH has implemented regulations on the clinical application of cutting-edge therapies such as stem-cell injections. In December 2011, the MH report of quality control in the implementation of stem cell clinical study and application indicated that stem cell clinical study and application activities not approved by the MH or SFDA should be discontinued (Table 2).

A total of 35 stem cell clinical trials have been performed in mainland China, 3 in Hong Kong and 22 in Taipei. As compared with the leading countries in the area of stem cell research, in China, the number of stem cell clinical trials is significantly smaller. The application of stem cells has mainly focused on hematopoietic stem cells and MSCs because of the type of stem cells used in clinical trials of hematological diseases, vascular diseases and diabetes mellitus.

Therefore, the gap between China and the United States as well as other leading countries in stem cell research is mainly characterized by innovation, regulation, and quality control. The review procedure and laws on stem cell clinical trials have not been established and the long-term mechanism needs to be improved.

### Tissue engineering

#### Construction of tissue-engineering research system in China

Tissue-engineering research in China started in the 1990s. In 1994, the Shanghai Science and Technology Committee established tissue-engineering research as a priority. In 1997, the topic of tissue engineering was officially recognized by the National Natural Science Foundation of China. In the same year, the first tissue-engineering laboratory, Shanghai Key Laboratory of Tissue Engineering Research, was established. In 1998, the national “973 Program” for basic research officially established tissue engineering as one of the research topics. In 2001 and 2002, the national “863 High-tech Research and Development Program” consecutively funded the application research and product development of tissue engineering. In 2001, the Shanghai Research and Development Center of Tissue Engineering, also known as the research and development center of tissue engineering in the biological field of the national “863 Program”, was established. In June 2012, 39 representative colleges and universities in the field of tissue engineering and RM cooperatively founded the collaborative innovation center for tissue engineering and RM (http://www.biotech.org.cn/news/news/show.php?id=13984, http://roll.sohu.com/20120630/n346960283.shtml).

In 2007, the SFDA announced the requirements for research and submission of tissue-engineered medical products (No. [2007]762 for medical devices). Requirements for the production environment of tissue-engineered technology in technical management specification for tissue-engineered tissue transplantation therapy (trial) were released by the MH in November 2009. At the same time, the MH issued the Management Specification for Class III Medical Device, New Technology for Cell Transplantation and Tissue-Engineered Tissue Transplantation. In 2013, China completed the registration and was granted the right to vote for International Standards Organization (ISO)/TC150/SC7 standardization activity as an active member country (P member) of the ISO/Technical Committee (http://www.gov.cn/gzdt/2013-04/05/content_2370611.htm) and Dr. Xiaobing Fu was appointed the chair of this committee in China. China was the 13th member and the first member in developing countries in Asia. Thus, the standardization task in the field of tissue engineering in China would officially go to the international stage for involvement in standardization activity of international relevant fields (Tables 3 and 4).

**Table 3:** Centers/key laboratories for stem cell biology and tissue engineering

Time	Unit
2001	Key laboratory for tissue engineering research, Institute of chemistry, Chinese Academy of Sciences
2003	Center for stem cell biology and tissue engineering, Sun Yet-Sun University
2003	Center of tissue engineering, Chinese Academy of medical sciences
2003	Center of tissue engineering, the Fourth Military Medical College
2005	Key laboratory for tissue engineering research, West China Medical University and Tsinghua University
2007	Center for biomedical materials and tissue engineering, Peking University
2008	Key laboratory for tissue engineering research, Polymer research institute of Tianjin University
2009	Key laboratory for tissue engineering research, Institute of basic medical sciences Academy of military medical sciences
2011	Zhejiang Key laboratory for tissue engineering and regenerative medicine founded by Zhejiang University

**Table 4:** Tissue engineering products approved for marketing by the SFDA

Company	Time	Name	Constitute	Application	Registration No.
Beijing Jieya Laifu Biotechnology	2006	J-1 allogeneic acellular dermal matrix	The donation of human body’s skin, removed the host immune rejection of all cells, while keeping intact the original structure with the same extracellular matrix	To repair oral mucosal defects, soft tissue defects.	SFDA (quasi-) word No. 2000 No. 346027, and No. 2006 3460430
	2010	AlloDerm	The donation of human body’s skin, removed the host immune rejection of all cells, while keeping intact the original structure with the same extracellular matrix	To repair defect of human dermal	SFDA (quasi-) word No. 2010 No. 3461247
Beijing Qingyuan Albert tissue engineering biological technology	2007	Rhino (acellular dermal matrix medical tissue patch)	Acellular dermal matrix derived from donated human skin that undergoes a multi-step proprietary process that removes both the epidermis and the cells that can lead to tissue rejection	To repair various causes of oral mucosa and soft tissue defect. Closing the wound, dental implantation, hernia repair, urethral	SFDA (quasi-) word 2004 No. 3460736
Chongqing Zongshen Junhui Biotechnology	2007	Artificial skin – gene transfection pigskin	The product performance and composition with fresh skin tissue taken from Bama miniature pig as basic material, and through the introduction of technology and gene transfection of CTLA4Ig gene research.	To repair burns and other trauma wound coverage, to promote wound healing, prevention of microbial infection	The Food Drug Armed (quasi) Word 2007 No. 3461287
Qidong Oriental Medicine Research Institute	2010	Acellular dermal matrix dressings	Pigskin as raw material, such as viral inactivation process with acellular prepared from a pig dermal extracellular matrix, is a porous three-dimensional network structure; mainly composed of collagen.	To repair superficial II degree burn wounds, donor site wounds, deep cut (cut) scab wound granulation wounds and other wounds.	SFDA (quasi-) word No. 2010 No. 3641111
Shaanxi Eyre skin Biological Engineering	2007	Tissue engineering skin	A bilayer artificial skin substitute: epidermal layer is composed of human epidermal cells, dermal fibroblasts from human and bovine collagen.	To repair deep II degree burn wound, not more than III degree burn wound 20 cm^2^ (diameter less than 5 cm)	The Food Drug Armed (quasi) Word 2007 No. 3461110
BiotechnologyYantai Zhenghai	2009	Skin repair film	Cattle skin tissue after treatment prepared by a series of acellular dermal matrix is composed primarily of collagen	To repair various causes dermal wound repair defects.	SFDA (quasi-) No. 2009, No. 3460425
	2009	Biofilm	Cattle skin tissue processed through a series of prepared acellular dermal matrix is composed primarily of collagen, collagen retains the unique three-dimensional structure	To repair a variety of causes dura (spinal) membrane defects	SFDA (quasi-) No. 2009, No. 3460602
	2009	Dental film	The leather prepared after a series of acellular dermal matrix, whose main ingredient is collagen, sterilized by radiation, one-time use	To repair various causes shallow intraoral soft tissue defect repair	SFDA (quasi-) No. 2009, No. 3460404
Biological Technology of Guangdong Grandhope	2007	General thoracic surgical repair film	Pig tissue membranes crosslinked epoxy chemical reagents and biochemical transformation of materials made of surgical repair	To repair chest wall, bronchial stump, diaphragmatic, visceral capsular defect, etc.	SFDA (quasi-) word No. 2007, No. 3461317
	2009	Sterile biological care record film	The pig offal film, by the addition of antigen and a series of biochemical treatment and viral inactivation and made	Skin burn, burn and trauma, skin defects	SFDA (quasi-) No. 2009, No. 3640426
	2011	Biotypes dura (spinal) membrane patch	Pig membranous tissue as raw material, processed into biotechnology, should be finished smooth side, the other side is allowed villous or striped or grid-like native structure	To repair hard brain (spinal) membrane defect	SFDA (quasi-) word 2008 No. 3460637
Beijing Datsing Bio-Tech	2011	Allogeneic bone grafts	Cadaveric bone usually obtained from a bone bank	Dental implants, to repair broken bones that have bone loss, and repair broken bone that has not yet healed	Fresh Armed State Drug Administration (prospective) ganxibaoNo.3460627 (2011)

#### Tissue-engineering studies

In 2007, the SFDA approved the first tissue-engineering product, ActivSkin, developed by the Fourth Military Medical University, Xi’an, which made China the second country in the world with the technology of artificial skin after the United States. Moreover, the country continued to develop tissue-engineered de-cellular dermal matrix, skin containing adipose layers, skin containing pigmentation, skin containing capillary-like network, skin containing hair follicles and dermal equivalents. In 2010, the SFDA approved bone-repair scaffolds developed by Fuzhai Cui, in Tsinghua University. This material has been used in 30,000 patients and was promoted to other parts of the world. Other products include tissue-engineered tendons, cartilage/bone, and neural tubes, etc (Table 4). The data could be collected on the website of the SFDA. Fundamental research or investigational use has been conducted for tissue-engineered oral mucosa, bladder, artificial liver and kidney, heart valve prostheses, cardiac patch, blood vessel, nerves, urethra, testes and thyroid glands. Thus, scientists in China have an interest in different fields and are involved in tissue-engineered grafts to repair and regenerate tissues and organ. In 2012, *Science* reviewed the development of tissue engineering in China; work from Xiaosong Gu at Nantong University, Yilin Cao at Shanghai Jiaotong University and Fuzhai Cui at Tsinghua University were highlighted for their outstanding contribution in this area [8].

#### Comparison with other research-advanced countries

The concept of tissue engineering was formally proposed by the US National Science Foundation in 1987. Moreover, the United States subsidized research projects for tissue engineering as early as 1988. Institutions involved are research institutes (National Aeronautics and Space Administration, the US Department of Energy, NIH), universities (Massachusetts Institute of Technology, Harvard Medical School, Georgia Institute of Technology, and University of California at San Diego), and enterprises (Sandoz, Organogenesis, Advanced Tissue).

The US FDA granted marketing approval to about 10 products including tissue-engineered skin and cartilage. In China, tissue-engineered skin has been approved, which marked the beginning of the industry for tissue-engineering products. The technical level of China in tissue engineering is basically geared to international standards, but the delay in tissue-engineered research and translational application in China stems mainly from regulations and product standards. With respect to tissue-engineering publications, China exceeded Germany, Japan and the United Kingdom as early as in 2000 and the gap with these countries has widened since 2008.

### Growth factors

#### Overview

The supplement of exogenous bioactive factors is important for accelerating wound healing and tissue regeneration. These bioactive factors include basic or acidic fibroblast growth factor (bFGF or aFGF), epidermal growth factor, nerve growth factor and bone morphogenetic protein. Some have been approved for clinical application as new drugs and approved with good clinical effects.

#### New growth factors

As early as in 1991, Chinese scientists published the first academic monograph that systematically addressed “growth factor and wound healing”. Since 1998, they have published results of a series of multicenter, randomized clinical trial about growth factors accelerating wound healing in *The Lancet* and other international journals [25]. The results indicated that bFGF and other active molecules play a key role in regulating tissue and organ repair and regeneration. About 10 growth-factor products have been approved by the SFDA and used in the clinic (Table 5).

**Table 5:** Growth factors for external using products approved for marketing by the SFDA

Company	Time	Products	Effect	Status
Zhuhai Essex Bio-Pharmaceutical	1998	Recombinant bovine basic fibroblast growth factor	Burn and scald wounds: including shallow II degree and deep II degree wounds, granulation wounds and inhalation injuries. * Acute wounds: bruises, contusions, combined injuries and cuts. * Surgical incisions: incisions of surgery, orthopedics, gynaecology (such as lateral episiotomy and cesarean incision), otolaryngology, urology and proctology. * Chronic wounds: diabetic ulcers, vascular ulcers, radiochemotherapy ulcers, bedsores, fistulas, residual wounds and cervical erosions. * Skin grafting: skin donor site, skin grafting site, skin flap handling. * Other applications: after plastic surgery, skin resurfacing, dermabrasion, nevus removal and laser therapy wounds.	SFDA Approval No.S10980077
	1999	Recombinant bovine basic fibroblast growth factor Eye Drops	* Various corneal defects and punctate keratopathy. * Recurrent punctate keratopathy in the shallow layer. * Mild or moderate dry eye. * Corneal operation and poor corneal healing after operation. * Geographic (or nutritional) herpes simplex keratitis. * Ballous keratitis. * Corneal abrasion, mild and moderate chemical burns.	SFDA Approval No.S19991022
	2005	Recombinant bovine basic fibroblast growth factor Gel	Treatment of various keratopathy, ocular traumas and foreign body removal. * Corneal transplantation and eye surface reconstruction. * Dry eye: especially dry eye due to corneal injury. * Cataract surgery (ECCE. Phaco etc.): restoration of endothelium and reduction of edema. * Corneal refractive surgery: repair damaged corneas before surgery, repair damaged nerves after surgery and treat postoperative dry eye	SFDA Approval No.S20050100
Beijing SL Pharmaceutical	2002	Lyophilized Recombinant Human Basic Fibroblast Growth Factor (rh-bFGF)	Chronic Cutaneous Ulcer and Burn Wounds	SFDA Approval No.S20020025
Nanhai Longtime Pharmaceutical	2004	Recombinant Human Basic Fibroblast Growth Factor for External Use	Chronic Cutaneous Ulcer and Burn Wounds	SFDA Approval No.S20040052
Shanghai Wanxing Bio-Pharmaceutical	2006	Lyophilized Recombinant Human Acidic Fibroblast Growth Factor For External Use	Chronic Cutaneous Ulcer and Burn Wounds	SFDA Approval No.S20060102
Guilin pavay gene Pharmaceutical	2002	Recombinant Human Epidermal Growth Factor Hydro Gel	Chronic Cutaneous Ulcer and Burn Wounds	SFDA Approval No.S20020111
Wuhan HITECK biological pharmaceutical	2006	Mouse nerve growth factor for injection	Peripheral nerve injury and peripheral neuropathy; Brain and spinal cord injury; Acute cerebrovascular disease, cerebral atrophy, Parkinson’s and Alzheimer’s disease.	SFDA Approval No.S20060051

Compared with China, the United States is more cautious about the clinical application of growth factors. Only recombinant human platelet-derived growth factor by Chiron was approved by the FDA in 1998 and is used for debridement healing and repair of advanced diabetic foot ulcers, severe burns, skin diseases, and bone and teeth defects. The recombinant human keratinocyte growth factor palifermin, by Amgen, was approved in 2004 and is used for treatment of severe oral mucositis caused by chemoradiotherapy. Therefore, the number is far less than that of growth factors approved in China.

Cell differentiation and dedifferentiation induced by growth factors are a “hot” topic in RM. In 2001, Chinese scientists published results of a series of in vivo and vitro studies dealing with epidermal cells dedifferentiating into epidermal stem cells in *The Lancet* and other international journals [26, 27], which addressed dedifferentiation as a new approach to make stem cells from skin. Particularly, the authors found that growth factors have a significant dedifferentiation effect on cells in vivo settings. Although the innovative theory immediately caused controversy between Chinese and foreign scholars, this groundbreaking thinking has great significance for the later practice of using active factors to reprogram adult cells into adult stem cells. These results offer direct evidence that the potential of plasticity and transdifferentiation of some adult tissue stem cells could lead to differentiation into other series of cell types without developmental correlation [28].

#### Other: gene therapy, therapeutic cloning and xenotransplantation

Gene therapy has an important place in neuron, cardiovascular and islet-cell regeneration. Self-developed recombinant hepatic growth factor is the first gene therapy product for cardiovascular disease and is now in a phase II clinical trial. In addition, pcD2/hVEGF121 gene therapy for peripheral vascular diseases has been approved by the SFDA for a special clinical trial, so China is the second country conducting such research after the United States.

“Therapeutic cloning” has always been listed as a national basic research program. It is divided into upper, middle, and lower research parts according to national strategic planning. Drs. Guoxiang Chen (Transgenic Research Center in Shanghai), Huizhen Sheng and Yilin Cao (Shanghai Second Medical University) are responsible for the upper, middle and lower research parts, respectively. With cloning, cells may be induced to differentiate into specific tissue and organs in vitro, such as skin, cartilage, heart, liver, kidney and bladder, then these tissues and organs are implanted into patients.

Xenotransplantation is being accepted gradually in addition to tissue engineering and stem cell technology. The first batch of inbred Wuzhishan mini-pigs with GT knockout (homozygous with 2 copies of GGTA1 gene deletion) were established by Deng and colleagues at the institute of animal husbandry. Pigskin excipients with reduced immunogenicity treated by several different means have been approved by the SFDA. In addition, patents have been applied for α-galactosidase–treated porcine heart valve, pericardium and ligament. Functional cells and various humanized organs for human transplantation may be obtained if the same method is applied in large animals. The immunological rejection of xenotransplantation would be overcome and result in functional organs.

## Conclusions

In 2012, a special edition of *Science* (sponsored supplement for Regenerative Medicine in China) gave a comprehensive snapshot of the development of RM in China, with particular emphasis on stem cells, tissue engineering and trauma. As the core scientific journal, *Science* echoed with positive comments for the achievements in China. For scientists in China, change and challenge in RM exists. Although its achievements are remarkable and impressed, China needs to strengthen the following aspects: (1) Sound administrative system, laws, technical specifications and guidelines. China has not yet established a truly effective management system for clinical application of somatic stem cells, and detailed regulatory rules are not clear. The standards for stem cell conservation, research, clinical trials, eligibility criteria for hospital and medical staff performing stem cell therapy, relevant instruments and devices remain to be determined. In 2009, the ministry of health put stem cell therapy into “third class medical technology”, which Mainly due to it involved the major ethical issues, and the security and effectiveness still need to further confirm by the specification of clinical trials research. In March 2015, the National Health Development Planning Commission and the China Food and Drug Administration jointly issued the “stem cell clinical research management approach (trial)”, which is first normative management documents about stem cell clinical research of China. Aim is restraint the action of stem cells in clinical research and to safeguard the rights and interests of the subjects, promote the healthy development of stem cell research [29]. The implementation should be in accordance with the guidelines for stem cell clinical translation released by the International Society of Stem Cell Research. Ethical norms and legal provisions should be strictly followed to constrain the application and clinical trials of stem cells. Emphasis should be paid on the protection of intellectual property, allowing healthy and orderly development of the study of RM. (2) Emphasis on training and retention of talented stem cell researchers. China has become an important member in RM competition. However, some prominent problems should be solved. Compared with international advanced levels, in China, the scale and overall level of stem cell and RM research still has a long way to go. The government must strengthen and improve the ethics construction and education in scientific research units, enterprises, hospitals to, enhance the management and cognitive level of medical staffs engaged in stem cell research. There is not much originality, especially not many innovative ideas and research directions that can lead the trend. China lacks worldwide influential scientists in the area of tissue regeneration (including stem cell, tissue engineering) and coordination among multi-disciplinary experts. (3) Reasonable allocation of resources and breakthroughs. China will use the limited funds to concentrate on RM research in inducing stem cell differentiation, synchronous repair and regeneration of multiple impaired tissues, construction of tissue-engineered major organs and driving tissue-engineered products from bench to bedside. Substantial progresses and breakthroughs have been made in further establishing and improving the rules and laws involved in RM and construction of a RM translational base. (4) Broad and deep international cooperation. China should continue to emphasize in-depth and practical cooperation with foreign well-known universities, scientific centers and key laboratories. The construction of programmed, systemic and open research teams at the national level can avoid the waste of resources and concentrate on advantages to overcome difficulties. (5) Diversification of investment and other new technologies. Stem cell technology is attracting scientists and entrepreneurs from various countries with its huge market potential and has tremendous business opportunities for stem cell industry. Some new technologies may bring breakthrough developments for RM, such as the effect of 3D biological printing technology on RM. Dr. Mingyan Xu, at Hangzhou University of Electronic Science and Technology, also the director of the research and development team for the Chinese biomaterials 3D printer, independently developed the first domestic biomaterial 3D printer. This printer has successfully printed small proportions of cartilage tissue of human ears and liver. This biomaterial 3D printer is characterized by a great variety of printed biomaterials, low incidence of cell damage, high printing precision and convenient operation. The development of nano-intellectual materials for RM is important. The intellectual biomaterials may induce molecular modifications of degradable materials, resulting in the interactions between cell integrins, induction of cell proliferation and differentiation and synthesis and assembly of extracellular matrix, thereby initiating the body’s regenerative system. For example, the new intellectual biomaterial can have the function of signal transduction and control the release of growth factors or drugs intelligently, thus inducing the regenerative repair of tissues and organs directly.

Finally, RM in China in the next 10 years is expected to achieve substantial progress in systemic and effective regulation as well as a management system for RM research, application and synchronous repair by inducing stem cells and regenerating a variety of impaired tissues. The construction of major organs by tissue engineering and large-scale application of tissue-engineered products will be completed. These achievements will bring hope to China improve healthcare and build a healthy society [30].

## Additional files

Additional file 1: Clinical trials about stem cells. (XLSX 8 kb) Additional file 2: Clinical trials about active factors. (XLSX 8 kb)

## Abbreviations

CAS: Chinese Academy of Sciences
FDA: Us Food and Drug Administration
iPS: Inducible pluripotent stem
MH: The Ministry of Health
MSCs: Bone-marrow mesenchymal stem cells
MST: The Ministry of Science and Technology
RM: Regenerative medicine
SFDA: The State Food and Drug Administration
